# EB病毒阳性弥漫大B细胞淋巴瘤诊断与治疗中国专家共识（2025年版）

**DOI:** 10.3760/cma.j.cn121090-20250729-00354-1

**Published:** 2025-09

**Authors:** 中华医学会血液学分会淋巴细胞疾病学组 中国临床肿瘤学会（CSCO）淋巴瘤专家委员会

## Abstract

EB病毒阳性弥漫大B细胞淋巴瘤（EBV^+^ DLBCL）是侵袭性弥漫大B细胞淋巴瘤的特殊亚型，预后不良。由于EBV^+^ DLBCL发病率低，我国临床医师对疾病的认识尚未统一，治疗缺乏规范性。为加强我国临床医师对EBV^+^ DLBCL的认识，提高诊断及治疗水平，推动开展多中心临床研究，中华医学会血液学分会淋巴细胞疾病学组、中国临床肿瘤学会（CSCO）淋巴瘤专家委员会组织相关专家，结合国内外最新研究进展，讨论并形成本共识。

## Abstract (en)

Epstein-Barr virus positive diffuse large B cell lymphoma（EBV^+^ DLBCL）is a rare subtype of diffuse large B cell lymphoma with a poor prognosis. Due to the low incidence of EBV^+^ DLBCL, the understanding of the disease has not been unified, and the treatment remains non-standardized. In order to strengthen the understanding of EBV^+^ DLBCL in our country, improve the diagnosis and treatment level, and help promote multi-center clinical research, the Lymphoid Disease Group, Chinese Society of Hematology, Chinese Medical Association and Lymphoma Expert Committee of Chinese Society of Clinical Oncology（CSCO）organized relevant experts to discuss and form this consensus on the diagnosis and treatment of EBV^+^ DLBCL, combined with the latest research progress in China and abroad.

EB病毒（EBV）阳性弥漫大B细胞淋巴瘤（diffuse large B cell lymphoma，DLBCL）是罕见的侵袭性B细胞淋巴瘤亚型。由于该病发病率低，我国临床医师对疾病的认识尚未统一，诊治尚不规范，为加强我国临床医师对EBV^+^ DLBCL的认知，提高诊断、鉴别诊断及治疗水平，中华医学会血液学分会淋巴细胞疾病学组、中国临床肿瘤学会（CSCO）淋巴瘤专家委员会组织相关专家参考国内外EBV^+^ DLBCL诊疗相关共识并结合国内外最新研究进展[1]，讨论并制定本共识以供临床参考。本共识已在国际实践指南注册与透明化平台（Practice guideline REgistration for transPAREncy, PREPARE）注册（注册号：PREPARE-2025CN822）。

一、定义及流行病学

EBV^+^ DLBCL是无已知免疫缺陷疾病或既往无EBV相关淋巴组织增生性疾病史（即除外慢性炎症相关DLBCL、淋巴瘤样肉芽肿病、原发渗出性淋巴瘤或浆母细胞淋巴瘤），且大多数肿瘤细胞核表达EBV编码RNA（EBV encoded RNA，EBER）的大B细胞淋巴瘤。目前国际上两种淋巴肿瘤分类中，2022年成熟淋巴瘤的最新国际共识分类（ICC）命名上保留WHO造血淋巴肿瘤第四版分类（WHO-4HAEM）中的EBV^+^ DLBCL（非特指，NOS），EBV阳性定义为>80％的肿瘤细胞表达EBER[2]；而WHO-5HAEM在命名上删除了第四版中“NOS”一词，直接命名为EBV^+^ DLBCL，该分类中EBV阳性的定义强调大多数肿瘤细胞应为EBER阳性，但并未设置肿瘤细胞EBER阳性阈值[3]。

EBV^+^ DLBCL可出现于各年龄阶段，发病率占全部DLBCL患者的10％～15％[1]，临床表现呈侵袭性，57％～70％诊断时为Ⅲ～Ⅳ期，半数以上患者诊断时合并结外受累，5年总生存（OS）率约为54.8％[4]–[5]。其中年轻（年龄小于50岁）患者的临床特征（疾病分期、结外受累情况、实验室检查等）总体上与老年患者类似，但预后显著优于老年患者[4],[6]。中国单中心66例患者的队列研究显示，中国患者呈现更具侵袭性的临床特征，2年无进展生存（PFS）率和OS率分别为40％和54％[7]。

二、病理诊断及鉴别诊断

EBV^+^ DLBCL可累及淋巴结和肺、消化道、皮肤、骨髓等结外部位，明确诊断需依靠病理活检。苏木精-伊红（HE）染色显示正常组织结构部分或完全破坏，异型大B细胞弥漫性浸润，其组织学模式可表现为多形性和单形性两种类型。在多形性病例中，转化的大细胞/免疫母细胞或霍奇金-里德-斯特恩伯格（HRS）细胞样肿瘤细胞散在分布于小淋巴细胞、浆细胞和组织细胞组成的反应性背景中，类似于富于T细胞/组织细胞大B细胞淋巴瘤。在单形性病例中，肿瘤细胞弥漫成片分布，背景中反应性免疫细胞少，与EBV阴性的DLBCL相似。一些病例中可见多形性和单形性模式混合存在。地图状凝固性坏死、血管中心性生长/破坏血管是其相对特征性改变，但并非总是存在。免疫表型上，肿瘤细胞表达广谱B细胞标志（包括CD19、CD20、CD22、CD79a和PAX5），常呈活化B细胞样免疫表型，MUM1阳性，BCL6表达不一，CD10几乎总是阴性。大多数病例CD30阳性，可呈部分弱表达至弥漫强阳性。在少数病例中，肿瘤细胞表达CD15，但缺乏经典型霍奇金淋巴瘤的其他免疫表型特征[8]。肿瘤细胞常表达PDL1和PDL2，尤其是在年轻患者中[9]。LMP1在大多数（>90％）病例中表达，而EBNA2仅在一部分（7％～36％）病例中表达，提示Ⅱ型EBV潜伏感染比Ⅲ型更常见。EBV原位杂交检测显示大部分肿瘤细胞EBER阳性。

EBV^+^ DLBCL的鉴别诊断具有一定挑战性，主要需与以下EBV^+^淋巴组织增生性疾病鉴别：①EBV^+^皮肤黏膜溃疡：常有已知的免疫缺陷/失调或免疫衰老，局限于结外部位，最常见于口腔黏膜、扁桃体、腭部及胃肠道，通常为单灶性、表浅且边界清楚的溃疡。EBV^+^大B细胞多少不一，分布于小淋巴细胞、浆细胞、嗜酸性粒细胞和组织细胞构成的多形性背景中，病灶最深边缘T小淋巴细胞呈带状浸润，亦可见凝固性坏死、血管中心性或血管破坏性生长。②淋巴瘤样肉芽肿病：患者亦无已知免疫缺陷/失调。但主要累及结外部位，最常见于肺部、中枢神经系统和皮肤，罕见累及淋巴结和骨髓。EBV^+^大B细胞分布于小淋巴细胞（主要为T细胞）、浆细胞和组织细胞背景中，一些病例亦可见凝固性坏死、血管中心性或血管破坏性生长。③免疫缺陷/失调相关的EBV^+^多形性B细胞淋巴增殖性疾病：患者有已知的免疫缺陷/失调病史，且浸润的B细胞可表现完整的分化谱系，包括小淋巴细胞、免疫母细胞、浆母细胞及浆细胞，免疫母细胞可不典型或类似HRS细胞，但缺乏大B细胞弥漫片状浸润，病灶内常伴有T细胞和组织细胞，结合病史及形态学可与EBV^+^ DLBCL鉴别。④慢性炎症相关DLBCL：该病罕见，其HE染色的形态与EBV^+^ DLBCL单形性模式相似，免疫表型亦重叠，但EBV潜伏感染形式主要为Ⅲ型。最常见于胸膜腔、骨骼（尤其是股骨）、关节和关节周围的软组织。发生于胸膜腔者，常有长达数年、数十年的脓胸病史；发生于骨和关节者，常有长达十年以上的慢性骨髓炎、金属植入史和慢性皮肤静脉溃疡病史。结合发病部位及病史可鉴别慢性炎症相关DLBCL与EBV^+^ DLBCL。⑤纤维蛋白相关大B细胞淋巴瘤（FA-LBCL）：是一种在慢性纤维蛋白沉积的自然或获得性解剖空间和部位偶然发现的大B细胞肿瘤，不经放化疗仍具有良好预后，尚无直接因FA-LBCL死亡的报道。HE染色表现为纤维蛋白包裹不典型免疫母细胞样大细胞，核分裂象和凋亡易见，但不侵犯周围正常组织，不形成肿块，结合此病理特点及临床表现可与EBV^+^ DLBCL鉴别。

三、检查、临床分期及预后分层

EBV^+^ DLBCL的诊断性检查与DLBCL相似，必要的检查包括：全身体格检查，尤其注意浅表淋巴结和肝、脾是否肿大，体能状态，有无B症状；实验室检查包括全血细胞检查、血生化检查、血清乳酸脱氢酶（LDH）水平以及乙型肝炎、丙型肝炎、HIV相关检测和脑脊液检查；影像学检查推荐PET-CT（优先推荐）或颈、胸、腹、盆腔增强CT检查及骨髓病理活检和骨髓细胞学检查，其中骨髓活检样本长度应在1.5 cm以上；其他检查还应包括心电图和心脏超声。条件允许时应完善潜在抗病毒遗传缺陷的检测，尤其是起病时可疑合并噬血细胞综合征或有长期发热病史的患者。

优先推荐采用PET-CT作为初诊EBV^+^ DLBCL患者分期及评估疗效的检查，推荐使用2014年Lugano修订版Ann Arbor分期系统进行疾病分期[10]。

EBV^+^ DLBCL患者的预后分层系统包括国际预后指数（International Prognostic Index，IPI）、Oyama模型和GELL模型。IPI评分包括年龄≥60岁、血清LDH>正常范围上限、ECOG评分2～4分、临床分期Ⅲ～Ⅳ期及结外受累部位>1个，每个指征1分。研究显示，IPI评分对EBV^+^ DLBCL的预后预测价值具有争议[11]–[12]。Beltran等[11]的研究显示，IPI评分0～2或3～5分患者中位OS期分别为64个月和5个月，差异有统计学意义（*P*＝0.03）。Oyama等[12]的研究表明，IPI评分不能有效预测EBV^+^ DLBCL患者的预后，并建立了Oyama模型，包括B症状及年龄>70岁2个因素，每个指征1分，将患者分为低危、中危和高危组，中位OS期分别为56.3个月、25.2个月和8.5个月（*P*<0.001）[12]。GELL评分则包含ECOG评分≥2分，结外受累部位>1个，血清白蛋白<35 g/L，血清LDH>正常范围上限及血小板/淋巴细胞比值>455，每个指征1分，将患者分为低危组（0分）、中危组（1～2分）和高危组（3～5分），用于预测患者的PFS及OS[13]。由于Oyama或GELL积分模型为小样本研究，均基于老年患者，预后分层未纳入年龄因素，目前在EBV^+^ DLBCL中的预测价值需要进一步评估。此外，与不良预后相关的因素还包括CD30表达量、PD-L1表达量及全血EBV-DNA水平等[7],[14]–[15]。对于EBV^+^ DLBCL患者的预后评估，共识专家组讨论建议同时进行预后评分，综合考量。

四、治疗

本专家共识推荐的治疗方案根据牛津证据分级系统（Oxford Center for Evidence-Based Medicine，OCEBM）对证据质量和推荐强度进行分级（表1）[16]。

**表1:** 2009版牛津证据分级系统（OCEBM）的推荐及证据强度等级[16]

推荐强度	证据强度	描述
A	1a	同质性好的多项随机对照试验（RCT）的系统评价
	1b	单个RCT研究（置信区间小）
	1c	显示“全或无”效应的任何证据
B	2a	同质性好的队列研究的系统评价
	2b	单个队列研究（包括低质量RCT，如失访率>20％）
	2c	基于患者结局的研究
	3a	同质性好的病例对照研究的系统评价
	3b	单个病例对照研究
C	4	病例系列报道（包括低质量队列研究及病例对照研究）
D	5	专家意见或评论

（一）初诊EBV^+^ DLBCL患者的治疗

初诊EBV^+^ DLBCL患者的治疗推荐参照初诊DLBCL，NOS的治疗方案进行。多个小样本回顾性研究显示，R-CHOP样治疗方案较CHOP样方案可显著提高EBV^+^ DLBCL患者的总反应率（ORR）、完全缓解（CR）率及生存数据[17]–[18]。Beltran等[17]的回顾性研究纳入33例EBV^+^ DLBCL患者，R-CHOP方案的CR率较CHOP方案高（59％对31％），R-CHOP方案的5年OS率也更高（54％对38％，*P*＝0.04）。且接受R-CHOP方案EBV^+^ DLBCL患者的OS率与EBV ^-^ DLBCL患者无显著差异[17]。因此，以R-CHOP方案为基础的方案仍为初诊EBV^+^ DLBCL患者的首选治疗方案（推荐强度B）。

POLARIX研究是Ⅲ期多中心随机对照研究，显示含CD79b抗体偶联药物（ADC）维泊妥珠单抗（polatuzumab vedotin）的pola-R-CHP方案较R-CHOP方案能更显著地提高初诊DLBCL，NOS患者的2年PFS率（77％对70％）[19]。由于该研究可纳入EBV^+^ DLBCL患者，虽然目前未公布EBV^+^ DLBCL亚型的分析结果，研究依旧提示pola-R-CHP方案有望成为EBV^+^ DLBCL初诊患者的首选治疗方案之一（推荐强度B）。

中山大学肿瘤防治中心蔡清清教授团队进行了塞利尼索联合R-CHOP方案治疗初诊EBV^+^ DLBCL患者的Ⅰb/Ⅱ期临床研究，18例EBV^+^ DLBCL患者接受塞利尼索（60 mg每周1次）联合R-CHOP方案治疗，16例可评估患者的CR率为75％，ORR为94％，显示出令人鼓舞的疗效和可接受的安全性[20]。因此，推荐塞利尼索联合R-CHOP方案作为初诊EBV^+^ DLBCL患者的治疗选择（推荐强度B）。

由于EBV^+^ DLBCL肿瘤细胞表面常高表达CD30或PD-L1，且上述分子表达与患者的不良预后相关。因此目前有小样本队列研究探索维布妥昔单抗（CD30-ADC，BV）或PD-1/PD-L1单抗治疗CD30或PD-L1高表达的DLBCL患者。Svoboda等[21]探索BV联合R-CHOP方案治疗CD30^+^的32例DLBCL患者，ORR为100％，CR率86％，2年PFS率和OS率分别为85％和100％，但该研究未具体显示EBV^+^ DLBCL亚组患者的例数和疗效。Manos等[22]的研究应用PD-L1单抗avelumab联合R-CHOP方案治疗28例初诊DLBCL患者，ORR为89％，CR率为89％，2年PFS率和OS率分别为82％和89％。该研究入组的3例EBV^+^ DLBCL患者的ORR为100％。另一项研究探索PD-1单抗Pembrolizumab联合R-CHOP方案治疗30例初诊DLBCL患者（包括2例EBV^+^ DLBCL患者），ORR为90％，CR率为77％，2年PFS率为83％，但没有公布EBV^+^ DLBCL患者的具体疗效[23]。根据上述证据，推荐特殊人群尝试联合BV（推荐强度D）、PD-1或PD-L1单抗（推荐强度D）。

（二）难治复发EBV^+^ DLBCL患者的治疗

目前仅针对难治复发EBV^+^ DLBCL队列的临床研究报道较少，总体治疗原则：①重新进行组织病理活检；②根据患者的体能状态、治疗线数及复发时间进行治疗分层；③专家组推荐治疗首选临床研究；④若无合适的临床研究，推荐难治复发EBV^+^ DLBCL患者参照难治复发DLBCL，NOS的治疗方案进行治疗（图1）。

**图1:**
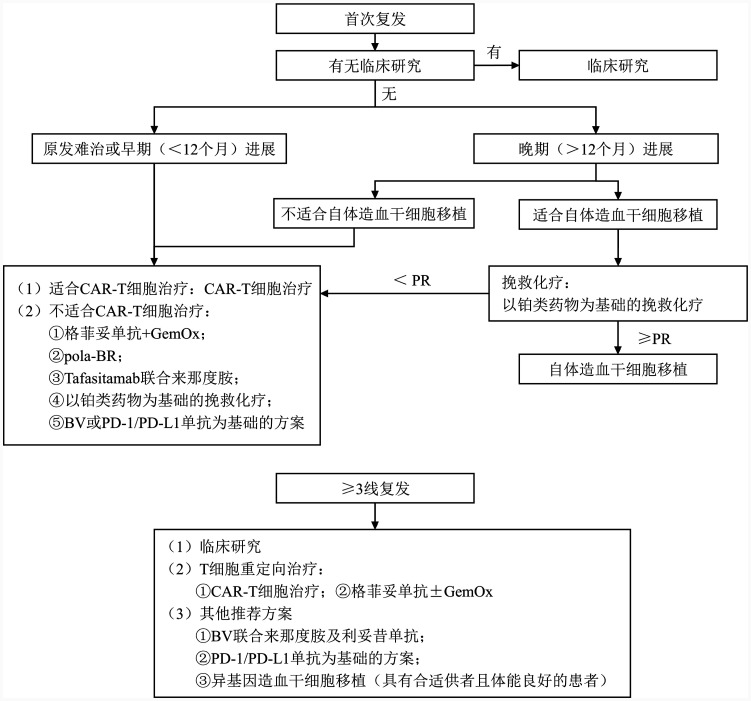
难治复发EBV^+^ DLBCL治疗流程图 **注** EBV^+^ DLBCL：EB病毒阳性弥漫大B细胞淋巴瘤；CAR-T细胞：嵌合抗原受体T细胞；GemOx：吉西他滨+奥沙利铂；pola-BR：维泊妥珠单抗+苯达莫司汀+利妥昔单抗；BV：维布妥昔单抗；PR：部分缓解

对于治疗后12个月内复发（早期复发）或原发难治患者，推荐合适的患者选择嵌合抗原受体T（CAR-T）细胞疗法。ZUMA-7研究提示，对于治疗后12个月内复发或原发难治的DLBCL患者，阿基仑赛较既往标准治疗能显著提高患者的OS率。该研究纳入2例EBV^+^ DLBCL患者，均随机分配至阿基仑赛组，但目前未公布EBV^+^ DLBCL亚型的具体疗效[24]。Lisocabtagene maraleucel（Liso-cel）具有和瑞基奥仑赛相同的CAR结构体，TRANSFORM研究比较了Liso-cel与既往标准治疗的疗效，同样提示对于早期复发或原发难治的DLBCL患者，Liso-cel较传统治疗更能使患者获益，但该研究未纳入EBV^+^ DLBCL患者[25]，共识专家组推荐CAR-T细胞疗法作为治疗后12个月内复发或原发难治EBV^+^ DLBCL患者的治疗选择（推荐强度B）。但国内学者曾报道EBV^+^侵袭性淋巴瘤在CAR-T细胞治疗后继发其他EBV相关淋巴增殖性疾病的病例，尤其是在合并潜在抗病毒遗传缺陷的患者中。因此，CAR-T细胞治疗后需定期监测EBV-DNA动态变化，可疑疾病进展时应完善病理评估[26]。对于不适合进行CAR-T细胞治疗的12个月内复发或原发难治的EBV^+^ DLBCL患者，共识专家组推荐采用格菲妥单抗联合GemOx方案（推荐强度D）[27]、pola-BR方案（推荐强度D）[28]或Tafasitamab联合来那度胺方案（不推荐用于原发耐药或“双打击”淋巴瘤患者）（推荐强度D）[29]。其他可选方案包括DHAP±R、ESHAP±R、ICE±R、GDP±R及GemOx±R等以铂类药物为基础的二线挽救化疗方案（推荐强度C）。若患者肿瘤细胞表达CD30或PD-L1，含BV[30]或PD-1/PD-L1单抗[31]的联合治疗方案也可作为治疗选择（推荐强度D）。

对于接受一线标准治疗后超过12个月首次复发的患者，若患者适合进行自体造血干细胞移植（auto-HSCT），且挽救治疗后疗效达到部分缓解及以上，推荐首选auto-HSCT而非CAR-T细胞治疗（推荐强度B）[32]–[34]。对于不适合进行auto-HSCT或挽救治疗疗效未达到部分缓解以上的患者，推荐接受CAR-T细胞治疗（阿基仑赛或瑞基奥仑赛）（推荐强度B），格菲妥单抗联合GemOx方案（推荐强度D）[27]，pola-BR（推荐强度D）[28]，Tafasitamab联合来那度胺（推荐强度D）[29]或GemOx±R方案治疗。特殊人群（肿瘤细胞表达CD30或PD-L1）亦可选含有BV[30]或PD-1/PD-L1单抗[31]的联合治疗方案（推荐强度D）。

对于经过二线标准治疗后复发的患者，推荐临床研究、CAR-T细胞治疗（阿基仑赛[35]或瑞基奥仑赛[36]）及格菲妥单抗±GemOx[27],[37]等治疗方案。虽然上述治疗的关键性研究均未纳入EBV^+^ DLBCL患者，共识专家组讨论后认为可作为治疗推荐（推荐强度D）。另外，对于不适合接受CAR-T细胞治疗且肿瘤细胞表达CD30的特殊人群，BV联合治疗方案可作为治疗推荐（推荐强度D）[38]。对于有合适供者、体能状态良好，且不适合进行CAR-T细胞或格菲妥单抗治疗，或上述治疗方案失败的多线复发EBV^+^ DLBCL患者，推荐进行异基因造血干细胞移植（推荐强度D）。

五、疗效评估

推荐根据2014版Lugano淋巴瘤疗效评估标准进行疗效评估（表2）[10]。疗效评估期间应定期监测患者全血EBV-DNA。

**表2:** 2014版Lugano淋巴瘤疗效评估标准[10]

分类	基于PET-CT的标准	基于CT的标准
完全缓解	D5PS评分1～3分，无新病灶，无骨髓浸润	靶病变（淋巴结或肿块）最大横截直径缩小至1.5 cm以下；无结外病变，肿大的器官缩小到正常，无新病灶，无骨髓浸润
部分缓解	D5PS评分4～5分，与基线相比，摄取减低。无新病灶，与基线相比，骨髓残留并摄取减低	与基线相比，最多6个靶病灶（最长直径×垂直于最长直径的短径）总和缩小>50％，肿大的脾脏较基线缩小>50％，无新病灶
病情稳定	D5PS评分4～5分，与基线相比，病灶摄取无变化。无新病灶，骨髓受累无变化	与基线相比，最多6个靶病灶（最长直径×垂直于最长直径的短径）总和缩小<50％，无新病灶
疾病进展	D5PS评分4～5分，与基线相比，病灶摄取增加。淋巴结或骨髓新发或复发高^18^F-FDG摄取病灶	淋巴结最长直径>1.5 cm；靶病灶最长直径×最长直径的垂直径增长>50％，淋巴结直径增长（<2 cm时增长≥0.5 cm；>2 cm时增长≥1.0 cm）。新发或复发脾脏肿大淋巴结，或新发或复发骨髓浸润

**注** D5PS：Deauville 5分评分系统；^18^F-FDG：^18^F-氟代脱氧葡萄糖

六、随访

对完成预定方案治疗的患者进行定期随访，前2年每3个月随访1次，随后3年每4～6个月随访1次，以后每年随访1次。随访内容包括病史、体格检查、血生化检查、全血EBV-DNA及影像学检查。影像学检查主要推荐颈/胸/腹/盆腔增强CT检查。
